# Book Notices

**Published:** 1892-11

**Authors:** 


### Book Notices.

A PRACTICAL TREATISE ON DISEASES OF THE SKIN. By Prof. John V. Shoemaker, A. M., M. D., Professor of Skin and Venerial Diseases in the Medico-Chirurgical College and Hospital of Philadelphia, etc. Second edition, 8vo, pp. 878. New York: 1892. D. Appleton & Co.

The first edition of this work was an acceptable addition to dermatological literature, and met with a ready sale. It soon became popular, rich as it was with the author's fine clinical observations. The JOURNAL reviewed it on its first appearance, and recommended it to its readers. This second edition shows careful revision, with many important additions. Important advances have been made in the mean time in several directions, especially in therapeutics, and the new work embraces the author's four years additional experience, and he is a close observer, and carefully records his observations. The whole work is written from the standpoint of actual experience; that is what makes all of Shoemaker's works so interesting and, we will add, so valuable. The profession is surfeited with theory; what they want is facts, and when Shoemaker tells us that such and such a drug will cure such and such a condition, for he has seen it done, one is satisfied. There has been so much disappointment with drugs sent out as cure-alls on "Unna's" say so, that practitioners are getting a little incredulous. Dr. Shoemaker gives us, in this work, the result of his carefully recorded experience with electricity as a remedial agent, which will doubtless revive the interest in electro-therapeutics, which had a boom in Texas a while back, but like some of her boom towns, it soon languished. The only objection the JOURNAL has to Shoemaker is his love of combinations—his polypharmacy; he does not seem to have much faith in single shot prescriptions. But most of his recipes are elegant combinations. This book contains a complete formulary for skin and venereal diseases, arranged alphabetically. This will be found very convenient for reference. On the whole, it is a most valuable book to have handy. Colored plates, taken from nature, illuminate the text, but they are too florid. Shoemaker is a trump; he is one of the best and most painstaking observers we have, and is doing a great deal for the honor and advancement of medical science. Long live Shoemaker!

DISEASES OF THE STOMACH. By C. A. Ewald, Professor of Medicine in the University of Berlin. Authorized translation (from second German edition, with special additions by the author), by Morris Manges, A. M., M. D., Attending Physician to Mt. Sinai Hospital dispensary, New York, etc.; with thirty illustrations. New York, 1892: D. Appleton & Co. Cloth.

This is one of the most valuable additions to medical literature of the year. It is a work of great practical importance, being devoted in part to the chemical analysis not only of contents, but of the tissues of the stomach. The chapter on "The practical value of the modern chemical test" alone is worth the price of the book. The work of translation was carefully revised and approved by the author, who made many additions; it is tantamount therefore, really, to a third German edition, which, we understand, is soon to appear. The book consists, it seems, of a series of lectures to practicing physicians, and is not intended as a text book for students. Particular attention is given to pathology, and the work is rich with the professor's clinical experience. We regard it as one of the most important works recently published.

---

THE MEDICAL AND DENTAL REGISTER-DIRECTORY AND INTELLIGENCER of Pennsylvania, New Jersey and Delaware (1892 edition); pp. 424; price, by mail, $1.25. George Keil, Publisher, 306 Chestnut street, Philadelphia.

This book contains a complete list of the National and State Medical and Dental Associations, with their officers and date of meetings, medical and dental colleges of the United States, and other very valuable material, medical and dental laws, hospitals, homes, etc., also the list of medical and dental practitioners, with their school and year of graduation, postoffice addresses, and office hours.

The work has been carefully compiled, and bears the impress of being thoroughly reliable in all its departments. It is well printed on good paper, nicely bound. A good book for reference, especially to publishers of current literature.

---

ADDRESSES AND ESSAYS. By G. Frank Lydston, M. D., Professor of the Surgical Diseases of the Genito-Urinary Organs and Syphilology, in the Chicago College of Physicians and

Surgeons, etc. Second edition, revised and enlarged. Published by Renz & Henry, Louisville, Ky.

We acknowledge with much pleasure the receipt of Professor Lydston's Essays, sent us with the compliments of the well known firm, Messrs. Renz & Henry, wholesale druggists, importers of new remedies, chemicals, chemical apparatus, etc., of Louisville, Ky. The members of the profession who receive this valuable work will appreciate the enterprise of this sterling firm who have already endeared themselves to the medical men of this country in many ways. They will send a copy free to any reputable physician.

---

THE AMERICAN TEXT-BOOK OF SURGERY FOR PRACTITIONERS AND STUDENTS. By Chas. H. Burnett, M. D.; Phineas S. Connor, M. D.; Frederick S. Dennis, M. D.; Wm. W. Keene, M. D.; Chas. B. Nancrede, M. D.; Roswell Park, M. D.; Lewis S. Pitcher, M. D.; Nicholas Senn, M. D.; Francis J. Shepard, M. D.; L. A. Stinson, M. D.; Wm. Thompson, M. D.; J. Collins Warren, M. D.; and J. W. White, M. D. Edited by Wm. W. Keene, M. D., LL. D., and J. William White, M. D., Ph. D. 8vo; pp. 1205. Philadelphia: 1892. W. B. Saunders, Publisher. Cloth, $7.

As we would naturally expect from the array of names above, —all men of eminence and acknowledged ability,—this work is a splendid one,—rich in the variety of subjects chosen, all tersely and practically treated. It covers the whole domain of surgery; and in light of our revolutionized pathology will supercede its predecessors, "Gross' System and Reynolds' System." Moreover, it is printed in clear, distinct type, which makes it easily read, notwithstanding the immense amount of material contained in the work. There is entirely too much of it for it ever to become popular as a text-book—student life is too short; but for a reference book for the physician in active practice it will prove a treasure indeed. There is a variety of illustrations, both as to quality and subject, embracing good, bad and indifferent, but all will answer their purpose. The work is bound to become a standard. It is to be regretted that it is sold by subscription only, as this puts it out of the reach of many.

---

TUBERCULOSIS OF BONES AND JOINTS. By N. Senn, M. D., Ph. D., Professor of Practice of Surgery in Rush Medical College;

Professor of Surgery in the Chicago Polyclinic; Attending Surgeon Presbyterian Hospital; Surgeon-in-Chief St. Joseph's Hospital; President of the American Surgical Association; President of the Association of Military Surgeons of the National Guard of the United States; Permanent member of the German Congress of Surgeons, etc. Illustrated with 107 engravings (seven of them colored). In one handsome Royal Octavo Volume. 520 pages. Extra cloth, $4 net; Sheep, $5 net; Half-Russia, $5 net. Philadelphia: The F. A. Davis Co., Publishers, 1231 Filbert street.

Professor Senn has made a more thorough and systematic study of this important branch of surgery than any man living; and being a close and accurate observer, and a good clinician, his statements and opinions are entitled to the highest consideration; he is authority on the subject. His observations and wide and varied experience with the treatment of this affection are recorded in this work, and will be read with much profit by even the best informed. It is a subject that has been slighted; touched on, perhaps, to the extent of a chapter or so in works on general surgery, and this is the only full and complete treatise extant, so far as we are aware, at least by an American author. In light of the revelations of bacteriology and the relation of germs to disease, the subject of tuberculosis of the joints acquires an increased interest. We predict for Dr. Senn's work an unprecedented sale.

---

TEXT-BOOK OF NERVOUS DISEASES, being a Compendium for the use of Students and Practitioners of Medicine. By Charles L. Dana, A. M., M. D., Professor of Nervous and Mental Diseases in the New York Post Graduate Medical School, and in Dartmouth Medical College; Visiting Physician to Bellevue Hospital; Neurologist to the Montefiore Home; ex-President of the American Neurological Association, etc. With 210 illustrations. Octavo, 524 pages, red parchment muslin, price, $3.25. William Wood & Co., New York.

Professor Dana's well known ability as a clinician, his long experience as a teacher and the habit of close and careful observation, entitle him to authority on the subject of nervous and mental disease, and his opinion to great weight. The book is timely too, as much more attention is being given to this branch of study by the general practitioner than formerly. The book is brought out with Wood's usual thorough attention to detail, and will doubtless be a popular seller.

---

DISEASES OF THE CHEST, THROAT, AND NASAL CAVITIES, including Physical Diagnosis and Diseases of the Lungs, Heart, and Aorta, Laryngology and Diseases of the Pharynx, Larynx, Nose, Thyroid Gland, and Œsophagus. By E. Fletcher Ingals, A. M., M. D., Professor of Laryngology and Practice of Medicine, Rush Medical College; Professor of Diseases of the Throat and Chest, Northwestern University Woman's Medical School; Professor of Laryngology and Rhinology, Chicago Polyclinic, etc. Second edition, revised and enlarged. 240 illustrations. Octavo, 700 pages, extra muslin, price, $5.00. William Wood & Co., New York.

The west is coming to the front. This work, written from the standpoint of western clinical experience, will make an interesting contrast with those heretofore published, mostly by Philadelphia and New York authors.

---

ESSENTIALS OF DIAGNOSIS, arranged in the form of questions and answers, prepared especially for students of medicine, by Solomon Solis-Cohen, M. D., Philadelphia, and Aug. A. Eshner, M. D., Philadelphia. With 55 illustrations, some of which are colored, and a frontispiece. 382 pages; price, net, $1.50. Philadelphia: W. B. Saunders, 913 Walnut street, 1892. This is a very handy book to have around.

---

THE JOURNAL has received from Geo. S. Davis, Publisher, Detroit, Michigan, a copy of Dr. Bulkley's neat little book on "Acne and Alopecia." Paper, 25 cents; cloth, 50 cents. This is a timely work, and Dr, Bulkley is authority. Acne is one of the most difficult diseases to treat satisfactorially, and a physician who is called on to treat the disease will find many suggestions here to help him out.

A TREATISE ON BRIGHT'S DISEASE OF THE KIDNEYS. Its Pathology, Diagnosis, and Treatment, with Chapters on the Anatomy of the Kidney, Albuminuria and the Urinary Secretion. By Henry B. Millard, M. A., M. D., New York. With Numerous Original Illustrations. Third Edition, Revised and Enlarged. PP. 322. Price—. New York. William Wood & Co., 1892.

